# *In vitro* transport of methotrexate by Drosophila Multidrug Resistance-associated Protein

**DOI:** 10.1371/journal.pone.0205657

**Published:** 2018-10-12

**Authors:** Agnes Karasik, András Váradi, Flóra Szeri

**Affiliations:** Institute of Enzymology, Research Center for Natural Sciences—Hungarian Academy of Sciences, Budapest, Hungary; Universidade do Minho, PORTUGAL

## Abstract

Methotrexate (MTX) is a widely used chemotherapeutic agent, immune suppressant and antimalarial drug. It is a substrate of several human ABC proteins that confer multidrug resistance to cancer cells and determine compartmentalization of a wide range of physiological metabolites and endo or xenobiotics, by their primary active transport across biological membranes. The substrate specificity and tissue distribution of these promiscuous human ABC transporters show a high degree of redundancy, providing robustness to these key physiological and pharmacological processes, such as the elimination of toxins, *e*.*g*. methotrexate from the body. A similar network of proteins capable of transporting methotrexate has been recently suggested to exist in *Drosophila melanogaster*. One of the key players of this putative network is Drosophila Multidrug-resistance Associated Protein (DMRP). DMRP has been shown to be a highly active and promiscuous ABC transporter, capable of transporting various organic anions. Here we provide the first direct evidence that DMRP, expressed alone in a heterologous system lacking other, potentially functionally overlapping *D*. *melanogaster* organic anion transporters, is indeed able to transport methotrexate. Our in vitro results support the hypothesized but debated role of DMRP in *in vivo* methotrexate excretion.

## Introduction

Adenosine triphosphate (ATP) binding cassette (ABC) proteins are considered as one of the most abundant protein families, existing in all phyla of life with 49 members in human and 56 in *Drosophila melanogaster* [1–3]. Most of the ABC proteins are membrane resident solute transporters, the eukaryotic transporters being exclusively exporters [1]. Most of the eukaryotic ABC transporters specifically export a limited number of closely related molecules, but there are a few transporters with an extremely wide spectrum of structurally unrelated substrates. Some of these promiscuous ABC transporters are involved in the multidrug resistance phenotype of cancer cells and are the major obstacle of cancer chemotherapy [4]. Methotrexate (MTX) is a widely used chemotherapeutic agent. It is an antifolate that effectively inhibits dihydrofolate reductase [5] catalyzing the formation of 5,6,7,8-tetrahydrofolate, which is essential for the biosynthesis of purines, thymidylate, and several amino acids. Thus, methotrexate inhibits the *de novo* synthesis of purine and pyrimidine bases and halts DNA, RNA and protein synthesis. It is used as a chemotherapeutic agent in various cancers such as leukemia, lymphoma, breast and lung cancer and osteosarcoma [4]. Due to its immune system suppressant effect, methotrexate is used in autoimmune diseases, such as Crohn’s disease, psoriasis, ulcerative colitis and multiple sclerosis. Methotrexate is first-line therapy for rheumatoid arthritis [6]. It also has potential as an antimalarial drug [7]. Methotrexate is a substrate of the most important human ABC proteins that can confer a multidrug resistant phenotype, *i*.*e*. ABCG2 [8, 9], ABCB1 [10] and ABCC transporters [11–13], which often hamper chemotherapy, or affect disease treatment. On the other hand, the physiological role of these transporters in eliminating toxic compounds from the body is of utmost importance. Multidrug ABC transporters often have overlapping substrate specificity and redundant tissue distribution, that provides sufficient chemoimmunity for the organism [14]. For instance, Abcc2, Abcc3, and Abcg2 together mediate the rapid elimination of intravenously administered MTX in mice and can compensate for each other's [15]. Drug-drug interactions, co-treatment with inhibitors of ABC proteins and specific mutations or polymorphisms in patients can influence the pharmacokinetics of methotrexate, resulting to increased toxicity or altered efficacy of treatment [16]. Investigation of such complex interactions requires efficient model systems, such as *D*. *melanogaster*.

Drosophila Multidrug Resistance-associated Protein (DMRP) is the only orthologue of all the “long” human C type ABC transporters (ABCC1, 2, 3, 6 and 10) in *D*. *melanogaster*. Long ABCCs form a subset of ABCC transporters, characterized by a unique transmembrane domain organization. In the topology of the long ABCC transporters the standard ABCC transporter domain structure of two transmembrane domains (TMD) and two nucleotide binding domains (NBD) in the form of TMD_1_-NBD_1_-TMD_2_-NBD_2_ is expanded by an additional N terminal TMD_0_ domain. DMRP shows high sequence similarity to the long ABCCs [17]. Our previous work demonstrated its ability to transport established long ABCC substrates such as estradiol-17-ß-D-glucuronide, leukotriene C4, 5-(and-6)-carboxy-2',7'-dichlorofluorescein, calcein and fluo3 [18, 19]. Transport of the above substrates is inhibited by known ABCC inhibitors (probenecid, benzbromarone, indomethacin and MK571) [18]. DMRP recently has been reported to play a role in developmental resistance against mercury [20], which is consistent with the DMRP orthologue ABCC2 being capable of transporting HgCl3- in mice [21]. In addition, reduced expression of endogenous DMRP has been shown to correlate with decreased secretion of the chemotherapeutic agent and ABCC1 substrate, daunorubicin, in the main insect excretory organ, the Malpighian tubules [22]. These data support the putative functional similarity of DMRP and long ABCC transporters *in vivo*. Methotrexate severely decreases female fecundity in Drosophila melanogaster and leads to serious developmental abnormalities in the offspring [23]. It also negatively influences the fitness of *Daphnia magna* through the marked loss of global DNA methylation [24]. Methotrexate has been shown to be excreted in the Malpighian tubules of *D*. *melanogaster* [25, 26]. Upregulation of dMRP expression by either chronic dietary MTX exposition or pharmacological induction, correlated with increased MTX secretion in the Malpighian tubules [25, 26]. Furthermore, established ABCC and DMRP inhibitors decreased tubular MTX secretion [25]. These data suggest a putative role of DMRP in the elimination of the toxic antifolate compound, MTX, via secretion by the Malpighian tubules. In response to chronic methotrexate exposure, a complex interaction of *dMRP* and two other organic anion transporters was revealed in *D*. *melanogaster*. However, indirect evidence with limited factors controlled, hinted that *dMRP* might not be a key determinant in the elimination of methotrexate [27].

The recent publications prompted us to develop *in vitro* assay systems to answer the question: Can DMRP, expressed alone in a heterologous system without the other potentially functionally overlapping *D*. *melanogaster* organic anion transporters transport methotrexate?

## Materials and methods

### Materials

Restriction endonucleases and T4 ligase were obtained from Fermentas and New England Biolabs, Pfu polymerase was provided by Stratagene. Oligonucleotides were ordered from Metabion International AG and Biological Research Center of Szeged. [3H]methotrexate ([3H]MTX; 25.9 Ci/mmol) was obtained from Moravek Biochemicals. The anti-DMRP polyclonal antiserum pAB7655 was raised against a synthetic peptide corresponding to amino acids 209–222 of DMRP (ZYMED Laboratories Inc.) as described previously [17]. Secondary HRP-conjugated anti-rabbit antibodies were purchased from Jackson ImmunoResearch. Nitrocellulose membrane filters (HWAP00250) were obtained from Millipore and the scintillation fluid (Opti-fluor) from PerkinElmer. All other compounds were obtained from Sigma Aldrich. Methotrexate (MTX) was dissolved in DMSO, the final concentration of DMSO in the assay buffer was kept less than 0.1% in transport and less than 1% in ATPase experiments.

### Methods

#### Generation of loss of function DMRP mutant

Residues in the highly conservative lysine of Walker A motifs of the NBDs of ABC transporters play a crucial role in the binding and hydrolysis of ATP [28] and in the activity of the transporters [29–32]. Therefore, we used the N- and C- terminal Walker A mutant DMRP (K687M/K1349M) as a loss of function (negative) control. We have previously cloned the 8a 4b isoform of *dMRP* cDNA (SD07655) into pAcUW21L [17]. The double Walker A catalytic center mutant DMRP was generated by Quickchange site-directed mutagenesis [19].

#### Expression of wild type and loss of function DMRP in Sf9 cells

Recombinant baculovirus particles containing wild type (SD07655) or K687M/K1349M mutant *dMRP* cDNA were prepared as described previously [17, 19]. Cultured *Spodoptera frugiperda* insect ovarium cells (Sf9) were infected with the recombinant baculovirus particles and harvested 3-days post virus infection.

#### Preparation of inside-out vesicles (IOV)

The Sf9 cell membranes overexpressing the proteins of interests were isolated as described previously [33]. The modified Lowry method [34] was used to determine total membrane protein concentrations. Gel electrophoresis and immunoblotting were performed to determine DMRP expression in the IOV preparations using the anti-DMRP polyclonal antiserum pAB7655 as described previously [17, 19] and IOV preparations containing comparable amounts of DMRP were used for the functional assays [19].

#### ATPase activity measurements

The vanadate-sensitive ATPase activity was determined by the liberation of inorganic phosphate as described previously [17, 33]. Briefly, IOV containing total membrane protein of 30μg or 50μg of wild type or K687M/K1349M DMRP, respectively, were incubated at 37˚C in 150μl buffer (40 mM MOPS-Tris, pH 7.0, 0.5 mM EGTA-TRIS, 2 mM dithiothreitol, 50 mM KCl, 5 mM sodium azide, and 1 mM ouabain) in the absence or presence of MK571 or benzbromarone (BB) as indicated. The ATPase reaction was started with Mg^2+^ATP in indicated concentrations. The assay was stopped by 0.1 ml of 5% SDS after 5 minutes unless indicated otherwise. Liberation of inorganic phosphate was determined in a colorimetric reaction after 15 minutes. ATPase activity was calculated as the difference in P_i_ levels after the indicated incubation time *vs*. *after stopping* the reaction with SDS immediately. Vanadate-sensitive ATPase activity (later referred to as ATPase activity) was determined as the difference between values measured in the presence and the absence of 1.33 mM ortho-vanadate. The figures depict mean values of at least three independent experiments done at least in duplicates. Experimental values were fitted by Michaelis-Menten equation in Prism v6.0e (Graphpad) where it was applicable. Statistical analysis was performed using Prism v6.0e (Graphpad). The standard error of the estimate of mean value (S.E.M.) was depicted.

#### Vesicular transport measurements

Vesicular transport measurements with radio-labeled methotrexate in IO vesicles were performed using rapid filtration [17]. Briefly, isolated IO Sf9 membrane vesicles of 50μg total membrane protein, were incubated in the absence or presence of 4mM Mg^2+^ATP, unless indicated otherwise. Assay was done at 37°C in 150 μl transport buffer (6 mM MgCl_2_, 40 mM MOPS-Tris, pH 7.0, 40 mM KCl) in the presence or absence of MK571 and benzbromarone (BB) as indicated. Transport assay was stopped by 800 μl of ice-cold washing buffer (40 mM MOPS-TRIS, pH 7.0, 70 mM KCl), after 0.5 minutes, samples were immediately filtered through 0.45 μm pore size nitrocellulose membrane filters (Millipore). Filter-bound radioactivity was measured in scintillation fluid (Opti-fluor, PerkinElmer) using Wallac 1409 DSA scintillation counter after being washed twice with 5 ml cold washing buffer at the end of the assay. ATP-dependent transport was calculated by subtracting activity values obtained in the absence from those in the presence of Mg^2+^ATP. The Michaelis-Menten kinetic parameters of transport were calculated using Prism v6.0e (Graphpad). Data points in the figures depict mean values of at least three independent experiments done in at least duplicates. Statistical analysis was performed using Prism v6.0e (Graphpad). The standard error of the estimate of mean value (S.E.M.) is shown.

## Results

### Methotrexate stimulates the basal ATPase activity of DMRP

The solute transport of ABC transporters is fuelled by hydrolysis of ATP. Most ABC transporters perform intrinsic basal ATPase activity in the absence of their substrates in Sf9 cells. In general, the ATPase activity of ABC transporters is further enhanced by the addition of their substrates. Therefore, stimulation of ATPase activity by a compound is indicative of the molecule being a substrate. To test the capability of DMRP to transport methotrexate (MTX), we first investigated its effect on the DMRP mediated hydrolysis of ATP.

**Fig 1:**
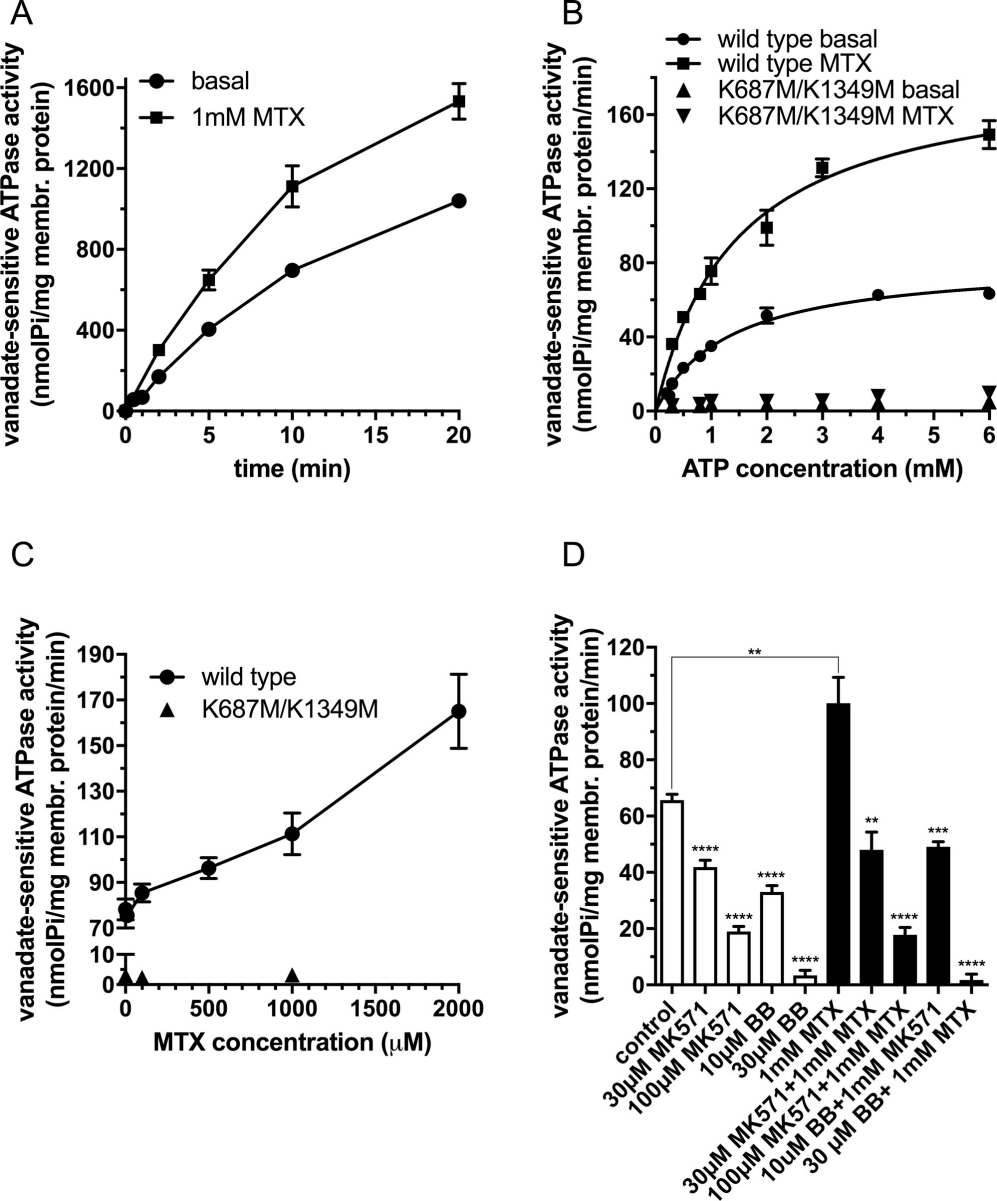
Vanadate-sensitive ATPase activity of DMRPs in Sf9 inside-out vesicles. **A:** Time course of ATPase activity measured in the presence of 3.3 mM Mg^2+^ATP at 37 ˚C using 30 μg total Sf9 membrane protein overexpressing wild type DMRP in the absence or presence of 1 mM MTX. **B:** ATPase activity as a function of Mg^2+^ ATP concentration measured at 37 ˚C for 5 minutes using 30 and 100 μg Sf9 IOV preparations overexpressing wild-type or K687M/K1349M DMRP, respectively in the absence or presence of 1 mM MTX. **C:** ATPase activity as a function of MTX concentration measured at 37 ˚C for 5 minutes in the presence of 3.3 mM Mg^2+^ ATP using 30 and 100 μg Sf9 IOV preparations overexpressing wild type or K687M/K1349M DMRP, respectively. **D:** ATPase activity of the wild type DMRP in the absence or the presence of organic anion inhibitors, MK571 and benzbromarone (BB), measured at 37 ˚C at 3.3 mM Mg^2+^ ATP concentration using 30 μg Sf9 IOV preparations overexpressing wild type DMRP in the absence (white bars) or in the presence (black bars) of 1 mM MTX. Figures depict mean values of at least three independent experiments done in at least duplicates. Of note, the standard error of the estimate of mean value (S.E.M.) is depicted for all of the data points in Fig 1, however error bars are often not visible due to the low scattering of the data. We used unpaired two tailed t-test for statistical analysis of data in 1D. Significance is indicated as *p≤0.05, **p≤0.01, ***p≤0.001 and ****p≤0.0001 between control and MK571 or BB inhibited samples in the absence or in the presence of 1mM MTX.

First, we investigated the time course of the ATPase activity in the absence and in the presence of 1mM MTX in inside-out vesicles prepared from Sf9 cells overexpressing wild type DMRP (Fig 1A). We detected a significant stimulation of the ATPase activity by methotrexate. We found that up to 5 minutes the ATPase activity showed a quasi linear correlation with time both in case of basal and methotrexate-stimulated ATPase activity. Therefore, in the following experiments we used 5-minute incubation time to measure the initial rate of ATP hydrolysis in the presence and absence of MTX.

Second, we investigated the DMRP dependent ATP hydrolysis on Mg^2+^ATP concentration in the absence or the presence of 1mM MTX (Fig 1B). In case of the double catalytic center mutant K687M/K1349M DMRP we did not detect notable ATP hydrolysis neither in the absence nor in the presence of MTX. However, we detected a high basal ATPase activity, and a significant methotrexate dependent stimulation of ATP hydrolysis for wild type DMRP. Both basal and methotrexate stimulated ATPase activities showed saturation kinetics with the parameters of 83.3+/- 3.1 and 186.3+/- 9.8 pmol Pi/mg membrane protein/min for the initial rate of ATP hydrolysis and 1.38+/-0.14 and 1.50+/-0.17 mM ATP for K_M_ for ATP, respectively. Next, we investigated ATPase activity of DMRP as a function of methotrexate concentration (Fig 1C). The rate of ATP hydrolysis showed a quasi linear correlation with MTX concentration in the entire concentration range. Due to the limited solubility of the compound we were not able to investigate ATP hydrolysis at MTX concentrations higher than 2mM. Therefore, we could not determine the kinetic constants for MTX in ATPase activity. But our data suggested a low affinity interaction of methotrexate with DMRP that stimulated ATP hydrolysis at high extent on a concentration dependent manner. Next, we investigated the ability of the established ABCC protein inhibitors benzbromarone (BB) and MK571 on the ATP hydrolysis of wild type DMRP in the presence or absence of 1 mM MTX (Fig 1D). We found that both benzbromarone and MK571 inhibited the basal and the methotrexate-stimulated ATPase activities of DMRP in a concentration dependent manner at concentrations generally applied to inhibit ABC transporter function in *in vitro* assays.

### Methotrexate is transported by DMRP

The chemotherapeutic agent and immunosuppressant drug methotrexate stimulated the ATPase activity of DMRP significantly (Fig 1) indicating an interaction of MTX and DMRP. To test directly whether methotrexate is a DMRP substrate we investigated the transport of radioactively labeled MTX in inside-out Sf9 membrane vesicles overexpressing wild-type and catalytic center mutant DMRPs.

**Fig 2:**
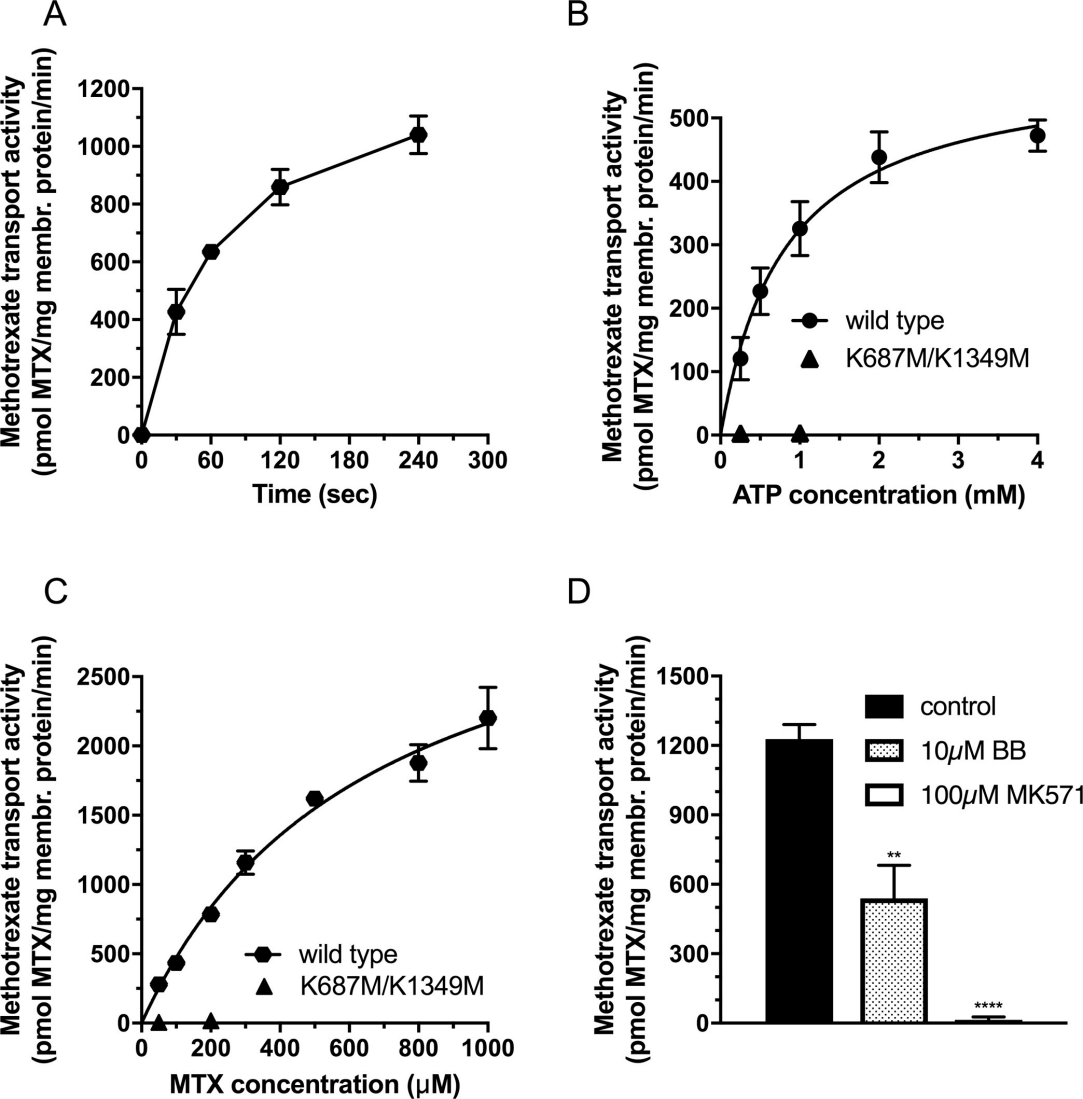
Methotrexate transport activity of DMRPs in Sf9 inside-out vesicles. **A:** Time course of MTX transport measured in the presence of 4mM Mg^2+^ATP at 37 ˚C using 50 μg total Sf9 membrane protein overexpressing wild type DMRP at 100μM MTX concentration. **B:** MTX transport activity as a function of Mg^2+^ ATP concentration measured at 37 ˚C for 0.5 minutes using 50 and 100 μg Sf9 IOV preparations overexpressing wild type or K687M/K1349M DMRP, respectively, at 100μM MTX concentration. **C:** MTX transport activity as a function of MTX concentration measured at 37 ˚C for 0.5 minutes in the presence of 4mM Mg^2+^ ATP concentration using 50 and 100 μg Sf9 IOV preparations overexpressing wild type or K687M/K1349M DMRP, respectively. **D:** MTX transport activity of the wild type DMRP in the absence or in the presence of organic anion inhibitors, MK571 and benzbromarone (BB), measured at 37˚C at 4mM Mg^2+^ ATP concentration using 50 μg Sf9 IOV preparations overexpressing wild-type at 300μM methotrexate. The figures depict mean values of at least three independent experiments done in at least duplicates. Of note, the standard error of the estimate of mean value (S.E.M.) is depicted for all of the data points in Fig 2, however error bars are often not visible due to the low scattering of the data. We used unpaired two tailed t-test for statistical analysis of data in 2D. Significance is indicated as *p≤0.05, **p≤0.01, ***p≤0.001 and ****p≤0.0001 between control and MK571 or BB inhibited samples.

First, we investigated the time course of methotrexate transport in the presence of 100 μM MTX in inside-out vesicles prepared from Sf9 cells overexpressing wild type DMRP (Fig 2A). We found a significant ATP-dependent MTX uptake to the IO vesicles. Tracer uptake was quasi linear up to 0,5 minutes, therefore, in the following experiments we used half-a-minute incubation time to measure the initial rate of MTX transport. Next, we investigated the uptake of the labeled MTX as a function of Mg^2+^ATP concentration at 100 μM MTX concentration (Fig 2B). In case of the double catalytic center mutant K687M/K1349M DMRP we did not detect notable MTX uptake. However, we detected a significant Mg^2+^ATP dependent methotrexate uptake with wild type DMRP. MTX accumulation showed a saturable function with the K_M_ for ATP at 809+/-119 μM ATP and V_max_ at 586+/-30 pmol MTX/mg membrane protein/min. The Michaelis-Menten constant for ATP for the MTX transport corresponded relatively well to that of the ATP hydrolysis. Next, we investigated the uptake of the radioactively labeled MTX as a function of MTX concentration (Fig 2C). We found no notable methotrexate transport for the K687M/K1349M mutant DMRP. In contrast, wild type DMRP showed high transport activity with saturable function of MTX concentration. By fitting the Michaelis-Menten equation we obtained the kinetic parameters of the apparent K_M_ as 660+/-94 μM MTX and Vmax as 3593+/-264 pmol MTX/mg membrane protein/min.

Next, we investigated the effect of known ABCC transporter inhibitors and established DMRP inhibitors, benzbromarone (BB) and MK571 [18], on the methotrexate transport activity of wild type DMRP (Fig 2D). At 300 μM MTX concentration 10 μM benzbromarone and 100 μM MK571 inhibited the DMRP mediated MTX transport to 44+/-12% and 0.5+/-1.7% residual transport activity, respectively, compared to that of the control (Fig 2B).

## Discussion

*In vivo* interactions of methotrexate with ABC transporters of *D*. *melanogaster* have already been reported. Tubular secretion of methotrexate was elevated upon chronic exposition to dietary methotrexate, which also resulted in the induction of the expression of additional organic anion transporters. The expression of *dMRP* was upregulated up to two thousand fold, in the Malpighian tubules and in the gut of fruit flies [25, 27]. Similarly, dietary exposure to piperonyl butoxide, a P450/monooxygenase inhibitor, altered the expression of several genes important in detoxification and organic anion transport, including a 250 fold upregulation of *dMRP* expression which was coincidental with significantly elevated MTX secretion by the Malphighian tubules [26]. Furthermore, inhibition of the transepithelial transport of MTX by DMRP inhibitors [18], MK571 and probenecid, also supported the putative role of DMRP in the efflux of methotrexate [25]. However, work on the MTX clearance in the Malpighian tubules reported complex interactions of three organic anion transporters *i*.*e*.: DMRP (*CG6214*) multidrug efflux transporter (MET, *CG30344*), and an organic anion transporting polypeptide 58Dc (OATP58Dc, *CG3380*), revealing that knockdown of either of these three genes alters the expression of at least one of the other two [27]. This work also presented a controversial indirect evidence hinting that *dMRP* might not be crucial in tubular secretion of methotrexate, based on a significant decline in methotrexate secretion in the MET and OATP RNAi lines accompanied by partially maintained dMRP mRNA levels. Yet, while changes in dMRP mRNA levels did not reach statistical significance in the OATP RNAi lines, it should be noted that the detected reduction was approximately 50%. Furthermore, the expression and localization of MET, OATP and DMRP at the protein level has not yet been investigated even though such a study would be indispensable to fully elucidate the *in vivo* role of these transporters in methotrexate secretion in D. melanogaster.

Here we present direct evidence that DMRP expressed in a heterologous expression system is capable of methotrexate transport at high capacity and thus provide evidence for methotrexate transport by one of the previously suggested candidate organic anion transporters. Our *in vitro* results and the recent compelling *in vivo* data strongly support the role of DMRP in the elimination of methotrexate in *D*. *melanogaster*.

Beyond it’s physiological and pathological relevance our work has biochemical significance as well. MRPs exhibit intrinsic ATPase activity in the absence of substrates, termed as “basal ATPase activity”. In this mechanism ATP hydrolysis is uncoupled from transport, as the substrate is missing, and ATP is hydrolyzed in a futile cycle. Interaction of the transporter with its substrate couples ATP hydrolysis to the transport of the substrate. In the majority of cases coupled ATP hydrolysis is higher than that of the basal ATPase activity, a phenomenon known as substrate stimulated ATPase activity. However, in some cases, substrates do not alter the rate of ATP hydrolysis, and seldomly they can even be inhibitory. The exact mechanism of uncoupled and coupled reactions is unknown. Coupled ATPase activity might be dependent on various factors, such as binding constants of the substrates to the low and high affinity substrate binding sites of the transporter or substrate-dependent differences in the cross talk between transmembrane domains and nucleotide binding domains exhibiting ATPase activity [19]. DMRP exhibits an extremely high basal ATPase activity. It is yet unclear if this elevated activity is the consequence of the presence of an endogenous substrate being transported in the Sf9 membrane representing a coupled mechanism or is it due to an uncoupled intrinsically high basal ATPase activity rate, characteristic of DMRP. Moreover, previous work found that the extremely high basal ATPase activity of wild type DMRP was found to be inhibited by its substrates [18, 19]. This phenomenon was explained by a hypothetical endogenous modulator (endogenous substrate or allosteric activator) in the Sf9 membranes [18]. According to this hypothesis an externally added substrate successfully competes with the putative endogenous substrate, but the rate of ATP hydrolysis coupled to the transport of the exogenous substrate is lower than that of the endogenous one, causing an apparent inhibition of ATP hydrolysis. Though the stimulatory effect of a few inorganic anions on the ATPase activity of DMRP has been already reported, the capability of DMRP to transport these drugs remained a question [18]. Our recent work showed evidence for the stimulatory effect of a substrate, estradiol-17-ß-D-glucuronide, on the ATPase activity of DMRP, but this effect was only present at extremely low ATP saturation, far below the physiological ATP concentration [19]. Unlike previously reported DMRP substrates, methotrexate significantly stimulated the basal ATPase activity of DMRP at physiologically relevant ATP concentrations. Thus, we presented here the first evidence that the extremely high basal ATPase activity of DMRP can be further stimulated by at least one of its transported substrates. Of note, the stimulatory nature of methotrexate on the basal ATPase activity of DMRP is compatible with the previously published endogenous modifier hypothesis, presumably showing a dominant effect of methotrexate over the putative endogenous modifier due to its higher potency to stimulate ATP hydrolysis. Taken all the known substrates in consideration DMRP is a prototypic transporter illustrating substrate dependent coupling mechanisms of both stimulatory and inhibitory nature.

In summary, our work demonstrates that DMRP, expressed alone in a heterologous system lacking the other potentially functionally overlapping *D*. *melanogaster* organic anion transporters, is able to transport methotrexate.

Moreover, we show that methotrexate can stimulate the intrinsically high basal ATPase activity of DMRP. Our *in vitro* results on the ability of DMRP to transport methotrexate are in line with *in vivo* experiments supporting that DMRP plays a key a role in the defense against methotrexate in *Drosophila melanogaster*.
